# Successful Treatment of a Patient with Drug-Refractory Rheumatoid Arthritis-Associated Interstitial Lung Disease with Upadacitinib: A Case Report

**DOI:** 10.3390/medicina59111960

**Published:** 2023-11-06

**Authors:** Yuuya Nishii, Masaki Okamoto, Yoshiaki Zaizen, Takashi Kojima, Takashi Nouno, Yoshiko Naitou-Nishida, Norikazu Matsuo, Hiroaki Takeoka, Motoko Ishida, Masataka Nakamura, Toru Masuda, Takafumi Tanaka, Tomoya Miyamura, Tomoaki Hoshino

**Affiliations:** 1 Department of Respirology, NHO Kyushu Medical Center, 1-8-1 Jigyohama, Chuo-ku, Fukuoka 810-0065, Japan; 2 Division of Respirology, Neurology and Rheumatology, Department of Internal Medicine, Kurume University School of Medicine, 67 Asahi-machi, Kurume 830-0011, Japan; 3 Department of Internal Medicine and Rheumatology, NHO Kyushu Medical Center, 1-8-1 Jigyohama, Chuo-ku, Fukuoka 810-0065, Japan

**Keywords:** interstitial lung disease, rheumatoid arthritis, Janus kinase inhibitor, upadacitinib

## Abstract

Insufficient evidence exists regarding the efficacy of Janus kinase inhibitors (JAKis), a class of targeted synthetic disease-modifying anti-rheumatic drugs (tsDMARDs), in the treatment of rheumatoid arthritis (RA)-associated interstitial lung disease (ILD). Herein, we present a case of RA-ILD refractory to previous treatments that exhibited favorable response to upadacitinib. A 69-year-old man, former smoker, was diagnosed with RA-ILD based on persistent symmetric polyarthritis, elevated C-reactive protein levels and erythrocyte sedimentation rate, reduced diffusing capacity for carbon monoxide/alveolar volume (D_LCO_ 69.9%), and bilateral ground-glass attenuation with traction bronchiectasis, predominantly in the lower lung lobe. Initial treatment with oral prednisolone and methotrexate was started; however, the patient showed worsening dyspnea, chest high-resolution computed tomography abnormalities, and decreased pulmonary function. The dose of prednisolone was increased, and methotrexate was shifted to tacrolimus; however, tacrolimus was eventually discontinued because of renal dysfunction. Subsequent treatment changes included abatacept followed by intravenous cyclophosphamide, but ILD activity continued to worsen and met the criteria of progressive pulmonary fibrosis. Approximately 4.5 years after the RA diagnosis, dyspnea, radiological abnormalities, and D_LCO_ improved following treatment switch to upadacitinib, one of JAKis. JAKi therapy may have potential as a treatment option for refractory RA-ILD.

## 1. Introduction

Rheumatoid arthritis (RA) is an inflammatory autoimmune disease characterized by joint inflammation and destruction, with a prevalence of approximately 0.5–1.0% in the adult population [1]. In addition to joint involvement, patients with RA can experience extra-articular manifestations, such as pulmonary and cardiovascular involvement [1,2]. Notably, the leading causes of death among patients with RA in Japan are pulmonary involvements and malignant diseases [2]. Interstitial lung disease (ILD) is known to develop in 1–67% of the patients with RA, with risk factors including male gender, older age, obesity, smoking, high titers of rheumatoid factor and anti-cyclic citrullinated peptide antibody (ACPA), elevated disease activity score 28-C-reactive protein (DAS28-CRP), and corticosteroid use [3,4,5,6]. Consequently, the development of therapeutic strategies for patients with RA-ILD is an important issue in RA, as patients with ILD exhibit significantly higher mortality rates than those without ILD [5,6,7,8].

The pathologic targets for RA-ILD drug therapy are classified as either pulmonary inflammation or fibrosis targets. Anti-inflammatory drugs include corticosteroids, immunosuppressants, and conventional, biological, and targeted synthetic disease-modifying anti-rheumatic drugs (cs, b, and tsDMARDs). However, limited reports exist regarding the effectiveness of the use of these drug therapies for ILD [9,10,11,12]. A randomized controlled phase 3 trial (INBUILD) provides evidence for the use of nintedanib in progressive pulmonary fibrosing ILD, including RA-ILD [13,14]. Moreover, a randomized controlled phase 2 trial (TRAIL1) for RA-ILD demonstrated that pirfenidone therapy improved the annual decrease in absolute forced vital capacity (FVC) as a secondary endpoint, although it did not meet the composite primary endpoint criteria (decline in FVC% from a baseline of 10% or more, or death) [15]. A major challenge in the use of anti-inflammatory therapy for patients with RA-ILD is the occurrence of adverse events, such as development of immunosuppression-induced infectious diseases and acute exacerbation of ILD [9,10,16,17,18]. Methotrexate (MTX) is an effective csDMARDs for the suppression of synovitis caused by RA as well as ILD in patients with subclinical or mild RA [11]. In fact, studies have indicated that MTX contributed to delaying the risk of developing ILD rather than being a risk factor [3,6,19]. Treatment guidelines by the American College of Rheumatology suggested that MTX is conditionally recommended for RA patients with clinically mild and stable respiratory disease and moderate-to-high disease activity [11]. However, patients with RA-ILD who have poor prognostic factors such as a radiological usual interstitial pneumonia (UIP) pattern and lower pulmonary function are not recommended for MTX treatment due to a risk of acute ILD exacerbation [6,20].

Previous retrospective studies of immunosuppressant treatment, including tacrolimus (TAC), azathioprine, mycophenolate mofetil, and bDMARDs, such as abatacept (ABA), tocilizumab, and rituximab, for patients with RA-ILD have demonstrated that these drugs are relatively safe [12,21,22,23,24]. A phase 3 randomized controlled trial for systemic sclerosis-associated ILD showed FVC improvement after tocilizumab or rituximab treatment, which were also expected to be effective for RA-ILD treatment [25,26]. Janus kinase inhibitors (JAKis), a class of tsDMARDs [27,28], have now been approved in Japan for patients with RA, particularly baricitinib, tofacitinib (TOF), filgotinib, and upadacitinib (UPA) [27,28]. However, evidence of the efficacy of JAKis in the treatment of RA-ILD remains insufficient [28,29,30,31,32,33,34,35]. Herein, we report a case of RA-ILD that was resistant to treatment with corticosteroids with intravenous cyclophosphamide (IVCY), TAC, and ABA that later showed a favorable response with UPA administration.

## 2. Case Presentation

**Figure 1:**
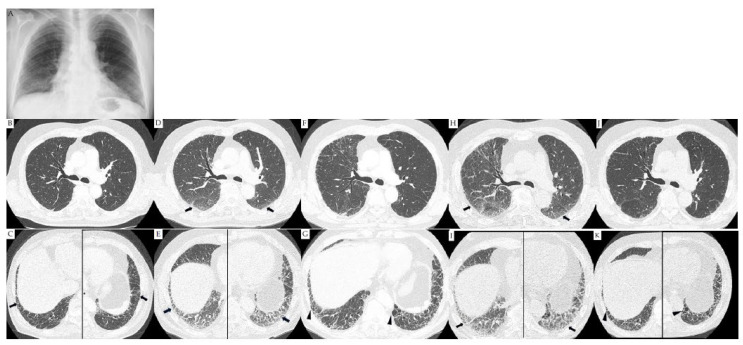
Images from chest radiography and high-resolution computed tomography. Chest X-ray showing ground-glass attenuation (GGA) and volume loss, predominantly in the bilateral lower lung field (**A**). Chest high-resolution computed tomography (HRCT) showing GGA with traction bronchiectasis (black arrows), predominantly in the bilateral lower lung lobes (**B**,**C**). Enlargement of the GGA with traction bronchiectasis (black arrows) in the bilateral lungs are observed (**D**,**E**). Reduced GGA and worsening traction bronchiectasis (arrow heads) are demonstrated (**F**,**G**). Greater enlargement of the GGA with traction bronchiectasis (black arrows) are exhibited (**H**,**I**). Reduced bilateral GGA (**J**,**K**).

**Figure 2:**
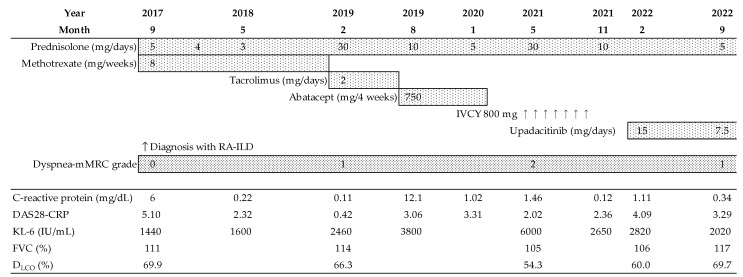
Clinical course. IVCY, intravenous cyclophosphamide; RA-ILD, rheumatoid arthlitis-interstitial kung disease; DAS28-CRP, disease activity score 28-C-reactive protein; KL-6, Klebs von den lungen-6; FVC, forced vital capacity; D_LCO_, diffusing capacity of the lungs for carbon monoxide.

A 69-year-old Asian male past smoker, who had a history of smoking three packs per day between the ages of 20 and 50 years and no comorbidities visited our hospital with persistent morning stiffness and symmetric polyarthritis, predominantly in the metacarpophalangeal joints of the bilateral hands for over 6 weeks; in June 2017, the patient had no respiratory symptoms such as dyspnea or cough. Vital signs on admission showed respiratory rate, 20 breaths/min; heart rate, 96 beats/min; blood pressure, 95/76 mmHg; and body temperature, 36.1 °C. Both his ACPA (0.7 U/mL) and rheumatoid factor (9.0 IU/mL) levels were negative. However, joint erosions by X-ray, synovial thickening and increased blood flow in musculoskeletal ultrasound, and elevated levels of C-reactive protein (CRP, 6.0 mg/dL) and erythrocyte sedimentation rate (57 mm/h) were observed. Based on the 2010 ACR/EULAR criteria (7/10), the patient was diagnosed with seronegative RA [36]. Further clinical data and radiological findings at the time of diagnosis are shown in Table 1 and Figure 1, respectively. Fine crackles on bilateral lungs and normal heart sounds were observed on auscultation. Hematological and laboratory examinations showed elevated levels of CRP and Krebs von den lungen-6 (KL-6, 1440 IU/mL). Pulmonary function tests demonstrated decreased diffusing capacity of the lung for carbon monoxide/alveolar volume (D_LCO_, 69.9%), although not restrictive or characterizing obstructive ventilatory impairment (FVC, 111.0% and forced expiratory volume in 1 s, 88.0%). A chest radiograph showed ground-glass attenuation (GGA) and volume loss, predominantly in the bilateral lower lung fields (Figure 1A). A chest high-resolution computed tomography (HRCT) scan showed GGA with traction bronchiectasis predominantly in the bilateral lower lung lobes at the time of RA diagnosis (Figure 1B,C). In accordance with global guidelines, the patient was diagnosed with RA-ILD with an alternative diagnosis of non-specific interstitial pneumonia pattern [37]. The clinical course of the patient is detailed in Figure 1 and Figure 2. 

The patient was Initially started on 5 mg of oral prednisolone (PSL) daily, 8 mg of MTX weekly, and a non-steroidal anti-inflammatory drug immediately upon diagnosis with RA-ILD. Approximately 1.5 years after the RA diagnosis, disease activity showed remission as the DAS-28-CRP of the patient decreased from 5.10 to 2.32 [38]. However, the patient developed grade 1 dyspnea based on the modified British Medical Research Council (mMRC) scale, enlargement of GGA with traction bronchiectasis on bilateral lungs observed on chest HRCT, and increased serum KL-6 levels (1440 IU/mL to 2460 IU/mL) (Figure 1D,E). Prior to the worsening ILD, the patient had no infection, and severe acute respiratory syndrome coronavirus 2 tested negative on polymerase chain reaction test. Worsening of the ILD was unknown. The dose of PSL was increased from 5 mg to 30 mg daily while MTX was replaced with 2 mg of TAC daily to stabilize ILD activity. However, treatment with TAC was eventually stopped because of renal dysfunction revealed by laboratory examination. Moreover, the patient experienced worsening arthritis approximately 2 years after RA diagnosis based on increasing CRP levels and DAS28-CRP. After 750 mg/week of ABA treatment, his DAS-28-CRP decreased to 2.02. However, we observed an increase in the mMRC grade from 1 to 2, an increase in the KL-6 level to 6000 IU/mL, and a decrease in D_LCO_ to 54.3%. HRCT images showed reduced GGA, whereas traction bronchiectasis worsened (Figure 1F,G). Approximately 3.5 years after diagnosis with RA, the ABA dose was changed to 800 mg/4 weeks IVCY, for seven cycles (total 5.6 g). Despite the shift in therapeutic plans, FVC and D_LCO_ continued to decrease while the area of GGA with traction bronchiectasis continually enlarged as observed on HRCT approximately 4 years after RA diagnosis (Figure 1H,I). This case was considered refractory to treatment and met the criteria of progressive pulmonary fibrosis based on global guidelines [14]. Daily administration of 15 mg UPA, a JAKi, was initiated approximately 4.5 years after RA diagnosis. At 29 weeks after starting UPA, the patient showed improvement in the mMRC grade (from 2 to 1), GGA on HRCT (Figure 1J,K), pulmonary function (FVC, 105 to 117%; D_LCO_, 54.3 to 69.7%), and DAS28-CRP (4.09 to 3.29). UPA was eventually tapered to 7.5 mg daily and PSL to 5 mg daily. At the time of publication, the patient showed no signs of worsening ILD.

For the present case report, written informed consent was obtained from the patient to publish this case and any images but approval by an institutional review board was not obtained.

## 3. Discussion

We reported a successful case of RA-ILD treatment using a JAKi in a patient whose disease was refractory to various treatments, including immunosuppressants and bDMARDs such as corticosteroids (with or without IVCY), TAC, and ABA. Scant evidence is available on the efficacy of JAKi therapy for ILD. However, this case suggests that JAKi may be a potential treatment option for RA-ILD. In our case, the patient had TAC-induced renal dysfunction which led to our selection of UPA, the only JAKi metabolized by the liver and not the kidney. The JAK/STAT pathways control cell homeostasis via signaling through numerous ligands, including cytokines [28]. Among the JAK family members, which include JAK1, JAK2, JAK3, and tyrosine kinase 2, JAK1 is found to be overexpressed in lung tissues and localized in inflammatory and epithelial cells in murine bleomycin-induced fibrosis models [29]. Histological analysis of human idiopathic pulmonary fibrosis samples revealed that JAK2 is mainly distributed in hyperplastic alveolar epithelial type II cells, fibroblasts, and the intima, as well as in the middle layer of the small pulmonary artery [30]. A previous study demonstrated that inhibition of the JAK2–STAT3 pathway suppressed interleukin-6 expression, transforming growth factor-β1 (TGF-β1)-mediated epithelial–mesenchymal transition, and differentiation of fibroblasts to myofibroblasts [30]. Several reports have also indicated that JAKis, including TOF, reduced lung fibrosis in animal models of ILD [28,29,30,31].

The efficacy of JAKi therapy has also been reported for rapidly progressive ILD with anti-melanoma differentiation-associated gene 5 (MDA5) antibodies [39,40,41]. In a retrospective cohort study using claims data from the Optum Clinformatics Data Mart, patients with RA treated with TOF had a lower risk of developing ILD than patients treated with other bDMARDs [32]. A previous retrospective study involving 43 patients with RA-ILD treated with JAKis showed that more than 80% of patients displayed improvement of pulmonary function and/or HRCT findings [33]. The study by d’Alessandro et al. reported a significant decrease in KL-6 after baricitinib therapy in patients with RA-ILD [34]. Regarding the efficacy of JAKi therapy in a refractory case of RA-ILD, Vacchi et al. reported a patient with ILD who was refractory to treatment with corticosteroids, hydroxychloroquine, MTX, and etanercept, who improved after treatment with TOF [35]. Kurasawa et al. reported the survival of three out of five dermatomyositis-associated ILD patients with anti-MDA5 antibodies who were refractory to high-dose glucocorticoids, cyclosporin A, and cyclophosphamide but improved after administration of TOF (10 mg/day), while all six historical controls who did not receive TOF died [39]. Because the present case who responded to UPA treatment but not to other bDMARDS, we believe that JAKis may have the potential to be a treatment option for patients with refractory connective tissue disease-associated ILD. In patients with RA-ILD, first-line therapy should include immunosuppressants and DMARDs to suppress arthritis and/or systemic inflammation, and antifibrotic drugs should be added if progressive pulmonary fibrosis develops [9,10]. Early addition of antifibrotic drugs should be considered in cases with a radiological UIP pattern [10]. The radiological lung non-specific interstitial pneumonia pattern in the present case, characterized by GGA predicted to be inflammatory lung involvement, decreased after JAKi therapy, but the traction bronchiectasis predicted as fibrosis remained. If radiological lung fibrosis worsens in the future for our patient, additional therapy with antifibrotic drugs is a viable option.

Venous thromboembolism (VTE) is a serious complication caused by JAKi therapy that should be noted. A meta-analysis of randomized controlled trial data in patients with immune-mediated inflammatory diseases has not provided evidence that support the current warnings of VTE risk for JAKi [42]. However, European Medicine Agency suggested that JAKi therapy for high-risk patients such as patients over 65 years or current or heavy smoker should only be performed if there are no suitable other treatment alternatives [43]. The present case was resistant to some other drug therapies and the most important prognostic factor was considered to be progressive fibrosing ILD [5,6,7,8]; thus, JAKi therapy was deemed appropriate.

## 4. Conclusions

We report a case of RA-ILD, refractory to immunosuppressants and bDMARDs, but with a favorable response to UPA. JAKi therapy shows promising potential as a treatment option for patients with refractory RA-ILD and should be investigated in further studies.

## Figures and Tables

**Table 1:** Clinical data at the time of diagnosis with rheumatoid arthritis.

Complete blood count		-	CRP	6.0	mg/dL
White blood cells	3900	/μL	Ferritin	389.8	ng/mL
Neutrophils	39.3	%	MMP3	268.9	ng/mL
Lymphocytes	39.1	%	KL-6	1440	U/mL
Eosinophils	1.0	%	Autoantibodies	-	-
Hemoglobin	12.2	g/dL	Antinuclear antibody	40	Index
Hematocrit	38.6	%	with homogeneous pattern	-	-
Platelet count	2.6 × 10^4^	/μL	Rheumatoid factor	9.0	IU/mL
ESR	57	mm/h	ACPA	0.7	U/mL
Laboratory data	-	-	Anti-RNP antibody	Negative	-
Total protein	7.5	g/dL	Anti-Ro60 antibody	Negative	-
Lactate dehydrogenase	237	IU/L	Anti-La antibody	Negative	-
Aspartate transaminase	21	IU/L	Anti-topoisomerase-1 antibody	Negative	-
Alanine transaminase	13	IU/L	Myeloperoxidase-ANCA	Negative	-
Total bilirubin	0.5	g/dL	Proteinase-ANCA	Negative	-
Blood Urea Nitrogen	21	mg/dL	Pulmonary function	-	-
Creatinine	0.87	mg/dL	FVC	111.0	%
Na	139	mEq/L	FEV1%	88.0	%
K	4.6	mEq/L	D_LCO_/VA	68.0	%

ESR, erythrocyte sedimentation rate; CRP, C-reactive protein; MMP3, Matrix metalloprotease; KL-6, Klebs von den lungen-6; ACPA, anti-cyclic citrullinated peptide antibody; ANCA, anti-neutrophil cytoplasmic antibody; FVC, forced vital capacity; FEV1%, forced expiratory volume per second; D_LCO_/VA, diffusing capacity of the lung for carbon monoxide/alveolar volume.

## Data Availability

The data presented in this study are available on request from the corresponding author. The data are not publicly available due to ethical considerations.
